# Flexible 3D Printed Conductive Metamaterial Units for Electromagnetic Applications in Microwaves

**DOI:** 10.3390/ma13173879

**Published:** 2020-09-02

**Authors:** Anna C. Tasolamprou, Despoina Mentzaki, Zacharias Viskadourakis, Eleftherios N. Economou, Maria Kafesaki, George Kenanakis

**Affiliations:** 1 Institute of Electronic Structure and Laser, Foundation for Research and Technology Hellas, 70013 Heraklion, Greece; d.mentzaki@iesl.forth.gr (D.M.); zach@iesl.forth.gr (Z.V.); economou@admin.forth.gr (E.N.E.); kafesaki@iesl.forth.gr (M.K.); gkenanak@iesl.forth.gr (G.K.); 2 Department of Materials Science and Technology, University of Crete, 70013 Heraklion, Greece; 3 Physics Department, University of Crete, 70013 Heraklion, Greece

**Keywords:** metamaterials, metasurfaces, 3D printing, Fused Deposition Modeling (FDM), Fused Filament Fabrication (FFF), microwave components, electromagnetic materials, polymers

## Abstract

In this work we present a method for fabricating three dimensional, ultralight and flexible millimeter metamaterial units using a commercial household 3D printer. The method is low-cost, fast, eco-friendly and accessible. In particular, we use the Fused Deposition Modeling 3D printing technique and we fabricate flexible conductive Spilt Ring Resonators (SRRs) in a free-standing form. We characterized the samples experimentally through measurements of their spectral transmission, using standard rectangular microwave waveguides. Our findings show that the resonators produce well defined resonant electromagnetic features that depend on the structural details and the infiltrating dielectric materials, indicating that the thin, flexible and light 3D printed structures may be used as electromagnetic microwave components and electromagnetic fabrics for coating a variety of devices and infrastructure units, while adapting to different shapes and sizes.

## 1. Introduction

Metamaterials and their two dimensional analogues, metasurfaces, are artificial materials with purposefully designed subwavelength, periodic elementary units, the meta-atoms, which provide controllable interactions with electromagnetic (EM) waves and enable exotic electromagnetic functions [1,2]. Metamaterials and complex media have been extensively experimentally evaluated and tested for real life applications and devices, such as antennas, sensors, splitters, isolators, modulators, electromagnetic shieldings and energy harvesters, in a large frequency range [3,4,5,6,7,8,9,10,11,12,13,14]. Their properties depend on both the meta-atoms’ architecture, and the constituent materials, which can be dielectrics, metals, semiconductors or 2D materials [15,16,17,18]. One of the most well-known and widely studied meta-atom is the metallic Split Ring Resonator (SRR). SRRs consist of metallic loops with gaps, and have been studied throughout the years in several variants [19,20].

For microwave applications, the dimensions of SRRs (and meta-atoms in general) lay in the range of mm and are commonly fabricated on printed circuit boards (PCBs) [20,21,22]. PCB technology has been significantly improved, and regarding the SRR production, exhibits very high printing resolution and high printing precision, resulting in the fabrication of high quality SRRs, in massive amounts, and thorough automated procedures with overall low production costs. On the other hand, PCB technology has distinct disadvantages. For example, in PCB technology, substrates are used, such as FR-4. The production of FR-4 is a complex procedure including several steps—drilling, cutting, laminating, chemical etching, oxidation, etc. Therefore, dedicated infrastructures and specialized personnel are required for the production of the FR-4 substrates. Furthermore, printing of SRRs on FR-4 requires complicated methods similar to those used for the FR-4 production. Thus, it is not considered as a straightforward process. Moreover, the FR-4-printed SRRs are strictly 2-dimensional, exhibiting almost not-flexibility, while FR-4 itself shows lossy behavior in the microwave regime, which impedes the performance of the SRRs printed on it. Lately, there has been a great interest in the construction of complex objects employing 3D printing technologies. 3D printing is an additive manufacturing procedure, wherein a complex 3D structure/object is constructed by stacking material layers. The shape of the desired object is drawn using a Computer Assisted Design (CAD) software. The produced CAD file is subsequently transformed to a corresponding code file, readable by the 3D printer (gcode), through appropriate software. Then, the 3D printer translates the gcode file, resulting to the construction of the desired object. The 3D printing process exhibits remarkable advantages; i.e., it is quick, cost-effective and user-friendly/eco-friendly (there is no need for handling chemicals and reagents) and it exhibits high resolution printing aspects.

To date, several 3D printing methods have been developed, namely, stereo-lithography, selective laser sintering, digital light processing, binder printing, inkjet printing and laminate object manufacturing [23,24]. Among them stands the Fused Deposition Modeling (FDM) or Fused Filament Fabrication (FFF) method, which is an effective 3D printing technique that uses long wires of thermoplastic materials called filaments. Filaments are heated above their melting point, and then they are extruded through a narrow nozzle. The nozzle moves in all xyz dimensions, according to the corresponding gcode. Thus the anticipated object is constructed layer-by-layer. The FDM process includes all the 3D printing advantages mentioned above. Moreover, FDM printers with exceptional printing characteristics (high printing accuracy, option of multiple printing filaments, heating sample beds, etc.) are commercially available, at reasonable prices, enabling the wide use of 3D printing technology.

Considering the above, the employment of the FDM method in the fabrication of flexible, stand-alone (no rigid substrate is necessary), fabric-like metasurfaces for microwave applications [25,26,27,28,29,30], appears to be a challenging, but promising idea. The challenge lies in the filament materials. Metamaterials suitable for microwave applications commonly involve metallic components, while most of the available thermoplastic materials for the FDM are fully insulating or have quite low conductivity values. Fabrication of metallic-like metasurface components via FDM is a not straightforward task and requires either additional fabrication steps for the metallization or careful selection of the designs and the FDM filaments. Nevertheless, compared to conventional PCB technology, construction of SRRs using FDM printing exhibits significant advantages: FDM printing is a single-step procedure; thus, the final SRRs are printed in one step. Moreover, no substrates are needed. The printed SRRs can be stand alone. Thus, any parasitic effects coming from the substrate are totally eliminated, improving the performance of the SRRs. Additionally, no dedicated infrastructure is needed. As shown later in the current study, household FDM printers and commercially available thermoplastics are used for SRR production. In addition, FDM printing allows for the construction of non-planar, 3-dimensional SRRs with improved performances (for example see reference [31]), and the use of thermoplastics enables the construction of flexible, shape-conformable SRRs, which can be perfectly fitted to in any surface.

In this context, we hereby present the fabrication and characterization of free-standing rectangular conductive SRRs, by employing the FDM technique. For the fabrication we used two different types of filaments resulting in the production of two separate SRR series. One of them was made using polylactic acid (PLA) filament, a widely known polymer material. Since PLA is insulating, the constructed SRR pieces were painted over with conductive silver epoxy. The other SRR series was built using a commercially available filament known as Electrifi. This filament consists of a polyvinylidene chloride (PVDC) matrix, in which copper (Cu) nanoparticles are included in a weight ratio of ~20% w/w, and therefore the filament exhibits significant electrical transport properties. Each SRR series consists of several species with varying geometries. Both SRR series were systematically characterized experimentally with the use of standard rectangular waveguides, and the measurements were corroborated with numerical simulations. Additionally, we evaluated the tunability of the structures’ responses to infiltrating dielectric material.

## 2. Materials and Methods

**Figure 1:**
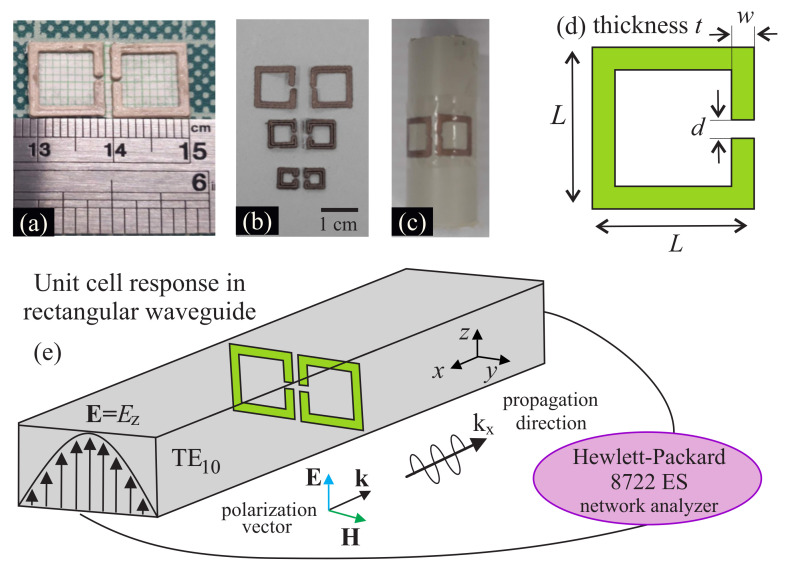
(**a**) Units of PLA coated with conductive Ag epoxy SRRs with opposing gaps attached on the top of a millimeter page. (**b**) SRR configurations of various dimensions made of Electrifi. (**c**) Electrifi SRRs (10 mm) attached to a plastic cylinder of 16 mm diameter. (**d**) Schematic of the SRRs unit and dimensions. (**e**) Characterization set-up; the meta-atoms are placed in the middle of the waveguide and the polarization of the field is along the side of the gaps. The size of the waveguide effectively defines the electromagnetic distance between the unit cells (periodicity).

We investigated the rectangular SRRs pictured in Figure 1. Initially we calculated the electromagnetic response of the structure using the three dimensional electromagnetic solver CST Suite. The details of the electromagnetic design are presented in Appendix A. The structures were designed using “Tinkercad”, which is free online 3D design and 3D printing software from Autodesk Inc. The structure consists of two SRRs oriented so that their gaps are next to each other, as shown in Figure 1. Said SRR configuration exhibits increased electromagnetic field confinement, which results in the high tunability of the response with relatively small changes in its environment [32,33,34]. Structures of variable length *L*, width *w*, thickness *t* and size of gap *d* (see Figure 1a,b) were produced. In Figure 1c one can see that the produced 3D printed SRRs are indeed flexible, since they are curved on a 16 mm diameter plastic cylinder, and they preserve their geometrical features. The distance between the adjacent SRRs is equal to the wire thickness *w*. For the purposes of the current study, we used a commercially available Makerbot Replicator 2x; a FDM printer; and two different, commercially available filaments as printing materials, i.e., the polylactic acid (PLA) and the Electrifi. Notably, 3D printing has been already used in fabrication of dielectric microwave metamaterials and components [35,36,37,38,39], and the conductive filaments have been also recently examined [40,41]. However, to the best of our knowledge, this is the first time that such a combination has been used for SRRs. To optimize printing results, we used appropriate printing conditions for each filament that are summarized in Table 1.

Two different series of SRRs have been constructed, one for each filament. Electrifi presents intrinsic conductivity due to the inclusion of the copper nanopatricles; the nominal value provided by the manufacturer is ~$1.6\times 10^{4}$ S/m. We covered the PLA-built SRRs with a thin layer (50μm) of the commercially available conductive silver epoxy (RS PRO number 186-3600) with an electrical conductivity of σ~$10^{5}$ S/m), which is typically used to repair PCB tracks or manually draw a simple circuit. The conductive Ag paste was applied using a thin painting brush for two times (2×). Between the first and second Ag paste layer the SRRs were left for 1 h, in air, so as the epoxy gets dry. The thickness of the Ag coating was estimated using a typical micrometer screw gauge, to be ~100 μm. The obtained SRRs consist of an insulating PLA core that is encapsulated into a shell of a metallic layer. At this point it should be noted that although PLA is a great alternative for conventional, non-degradable plastics based on fossil raw materials, it is also biodegradable. Following our proposed approach, when encapsulating PLA in a few hundreds microns of silver it is possible to insulate/protect PLA from the environment, and thus increase its *time of life*. Circular, Ag-plated SRR structures, have been already grown and studied regarding their microwave metamaterial properties [42], rendering the metal-coated, polymer-based SRR a promising design for microwave applications.

**Figure 2:**
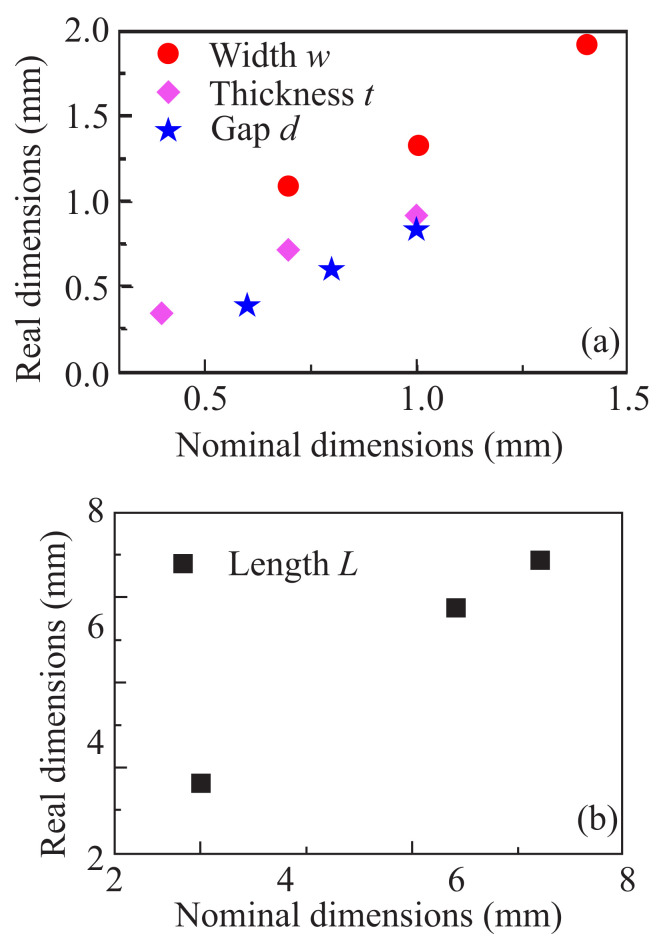
Measured versus nominal dimensions for PLA/Ag epoxy 3D printed SRRs, regarding (**a**) the width *w* (red circles), the thickness *t* (magenda rhombs) and the gap size *d* (blue stars) of the SRR while panel. (**b**) The corresponding values for the SRR length *L*.

Figure 2 shows the real dimensions of the PLA-built SRRs, with respect to their nominal values, imported to the CAD file. It is shown that the real length *L* is constantly larger than the corresponding CAD value (Figure 2b). On the other hand, real thickness, *t*; width, *w*; and gap, *d*, dimensions are lower than the nominal ones (Figure 2a). Furthermore, visual inspection of the gap (Figure 1) shows that the gap walls are not sharp-ended, but rather round. The rather high differences between nominal and real dimensions along with the not well-shaped gap, imply a sizable low printing resolution. Although the printing resolution of the printer was ~100 μm according to the manufacturer, in our study the printing resolution was estimated as around ~0.3–0.4 mm. In general, the printing resolution is affected by the nozzle diameter, the nozzle speed, the extrusion speed and the filament type. In the current study all the above parameters were chosen according to the PLA filament manufacturer guidelines. However, the rather low dimensions of the sample (~1 mm), in combination with the nozzle diameter (0.4 mm) probably contributed contradictorily towards the suppression of the final printing resolution. Regarding the printing quality, the printed SRRs are smooth, surface-clean and robust (Figure 1). The quality can be further improved by increasing the printing resolution. Furthermore, the samples are quite flexible, as shown in Figure 1e, in which 10 mm SRRs are attached to a cylinder of 16 mm diameter. Similar conclusions were extracted regarding the Electrifi SRRs.

The SRR components were placed on top of a thin piece of millimeter paper (see Figure 1a), which is totally transparent in the microwave regime; hence, they are considered to be electromagnetically free-standing. The electromagnetic response of each SRR pair was measured with the use of a standard rectangular waveguide (Figure 1e). In particular, we performed transmission measurements using an Agilent/Hewlett–Packard 8722 ES vector network analyzer (Agilent Technologies, Inc., Santa Clara, CA, USA). For the characterization we used waveguides of variable sizes with single mode frequency of operation that in total covered the range 3–16 GHz; in particular, we used the WR187, WR137, WR90 and WR62 waveguides. In the single mode operation the waveguide propagation mode was polarized along the small side of the rectangular cross-section, as seen in Figure 1e and explained in detail in the Appendix A. The SRR units under investigation were placed in the middle of the waveguide. Measuring the units in this way provides a ready, closed-system characterization of the electromagnetic response. As a reference, we have used the numerical study presented in Appendix A.

## 3. Results and Discussion

Having fabricated the samples, we proceed with the experimental electromagnetic characterization along with the numerical verification of the experimental findings. For the numerical calculations we used a full wave analysis. For each meta-atom under consideration we use the measured values assuming a deviation of 2% to account for structural mismatches, including possible deformations, rounded edges, thickness uniformity, etc. For the PLA/Ag samples we assumed a uniform material of conductivity $\sigma =10^{5}$ S/m and for the Electrifi $\sigma =1.6\times 10^{4}$ S/m, allowing in both cases a deviation of 0.5% about the nominal value. To simulate the rectangular waveguide we assumed surrounding metallic walls, while the fundamental mode was excited in the input port. Subsequently we calculated the scattering parameters (transmission).

**Figure 3:**
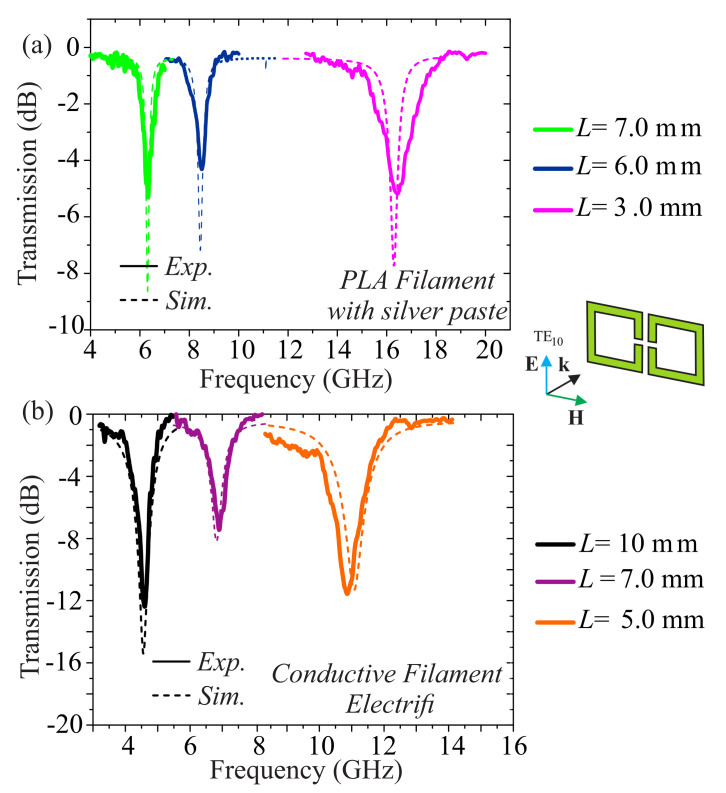
Electromagnetic investigation of the (**a**) PLA/Ag epoxy and (**b**) Electrifi built SRRs structure, experimental (solid curves) and numerical (dashed curves) transmission spectra. Cases of structures with variable length, *L* = 3–10 mm are presented. For all cases, the geometrical parameters are *w* = 1 mm, *t* = 0.4 mm and *d* = 0.6 mm as shown in Figure 1.

**Figure A1:**
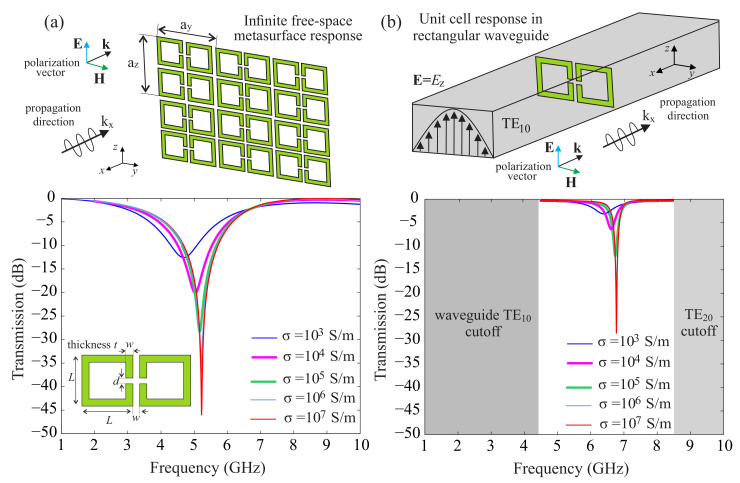
Numerical investigation: (**a**) Transmission spectra from a freestanding free-space, infinite, periodic metasurface, for various electrical conductivities. The side length of the SRR was L=6 mm, the width of the metallic frames was w=0.4 mm and the thickness of the metal was t=0.4 mm. The size of the gap was d=0.2 mm and the periodicity of the metasurface was $a_{y}=2\left(L+w\right)$ and $a_{z}=\left(L+w\right)$ along the *z* and the *y* axes. The incidence was normal and the polarization was along z axis, **E** = $E_{z}$. (**b**) Corresponding transmission spectra of the metasurface placed in a rectangular waveguide. The dimensions of the waveguide are a×b= 35 mm × 15 mm.

Figure 3a shows the experimental evaluation of the PLA/Ag epoxy SRR samples along with the numerical corroboration. In particular, we measured three unit cells with L=3, 6 and 7 mm. All SRR structures show well-defined, moderately sharp (−4 to −5 dB) resonances. The first thing to comment on is the level of the transmission dip, which was particularly shallow with respect to conventionally built metasurfaces [43]. This is a direct consequence of the produced material and the rectangular waveguide characterization. The decreased electrical conductivity exhibited by the 3D-printed PLA/Ag epoxy SRRs, in comparison to the PCB-built ones, led to the inductance of weaker currents in the meta-atoms. The conductivity decrease directly resulted in the reduction of the quality factors of the resonances, that is, the amplitude resonance transmission, along with a resonance broadening also accompanied by a shift to lower frequencies. A nelative numerical study is presented in the Appendix A. In particular, in Figure A1a the response of the free-space metasurface with variable conductivity $\sigma =10^{3}–10^{7}$ S/m was calculated. For values as high as $\sigma =10^{7}$ S/m, which is the case for pure metallic meta-atoms fabricated with, for example, PCB technology, the resonances are dip to −45 dB, whereas the corresponding metasurface made of a material of $\sigma =10^{4}–10^{5}$ S/m assumes resonances as low as −25 dB and poorer quality factors, yet efficient resonant behavior. At the same time, in Figure A1b it is shown that the waveguide characterization leads to an artificial degradation of the performance. We chose, however, to use this characterization method as it simplifies both the fabrication and the measurement, having in mind the analogy to the free space metasurface. As expected, the largest SRR pair showed resonance at ~6 GHz, while the smallest pair showed resonance at ~16 GHz, a result of the structures scaling. The numerical simulations successfully reproduced the resonance frequency, for all structures. The size of the SRR is the defining parameter for the position of the resonance. Other parameters that affect the resonance are the size of the gaps, the thickness of the samples and the width of the wires. The relevant results are presented in the Appendix B where it is shown that their effect on the resonance is relatively small. Some discrepancies are the results of various parameters such as the resolution of the 3D printing process, as discussed previously; the application of the silver paste which can result in non-uniformly thick shells with effectively lower conductivity; possible deformations; and the details of the electromagnetic characterization in the rectangular waveguides. The wriggles in the transmission spectra are associated with measurement noise.

We now turn to the SRR series made by Electrifi filament. Figure 3b presents the experimental and numerical characterization of the structures electromagnetic response. In particular, structures with L=5,7 and 10 mm were investigated. Once again, we observe that the unit cell’s physical upscaling leads to a shift of the resonance towards lower frequencies. Additionally, we observe that the resonances are well defined, sharp and relatively deep. Consequently, we see that although the conductivity of the Electrifi filament (~$1.6\times 10^{4}$ S/m) is orders of magnitude lower than that of cooper, it could be sufficiently high for certain microwave applications. The wriggles in the transmission spectra are associated with measurement noise.

**Figure 4:**
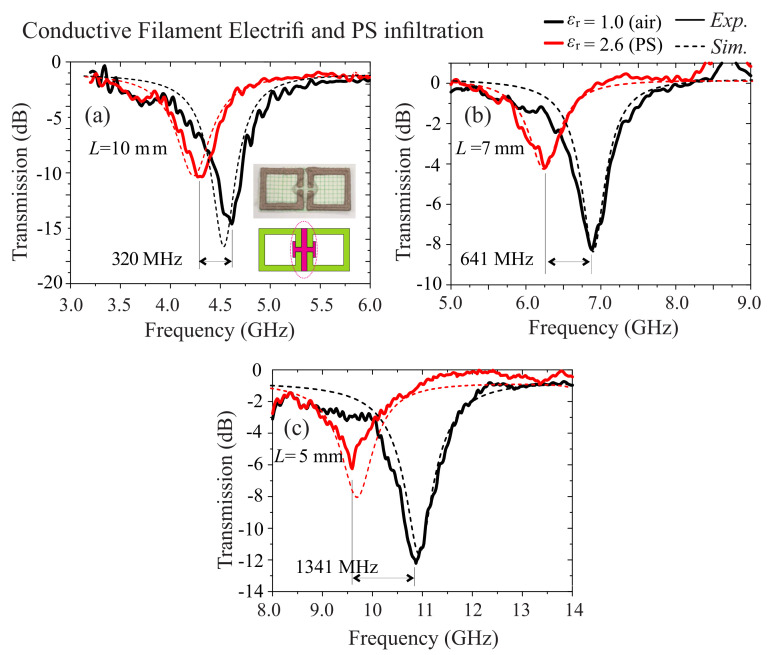
Tunability of the Electrifi SRRs with respect to the infiltrating polysterene ($\varepsilon_{r}$ = 2.6). Experimental (solid) and numerical (dashed) investigation of the transmission tunability in the rectangular waveguide for variable length: (**a**) *L* = 10 mm, (**b**) *L* = 7 mm and (**c**) *L* = 5 mm. The insets in (**a**) show a photo of the area of the infiltration for the sample *L* = 10 mm and a schematic of the averaged infiltration area in magenta, used for the numerical simulation.

Finally, the tunable electromagnetic response of the structure in the presence of an infiltrating material was evaluated. When an electromagnetic wave interacts with the SRR, alternating currents are excited along the elements while charge is accumulated in the adjacent elements. Consequently, the SRR behaves as an inductor-capacitor (LC) circuit with a characteristic resonance frequency $f_{0}$~$1/(2\pi \sqrt{LC})$. At the resonance a large confinement of the fields in certain areas of the meta-atom takes place—in particular in the area between the gaps and around them [32,33,34]. Placing a dielectric material in the high field position tunes the resonance. Thus, we evaluated the tunability of the SRR structures made by Electrifi filament. In particular, in the area of the facing gaps of the SRR structures, we gently added a droplet of polysterene (PS), diluted in toluene. After slightly heating, the toluene was evaporated and the PS was solidified into the SRR gap; the droplet spread around the area of the gaps. The polysterene exhibited a dielectric permittivity of $\varepsilon_{r}$ = 2.6 [44]. The experimental and numerical study for samples with L=10, 7 and 5 mm are presented in Figure 4. In the inset of Figure 4a we include a photo of the droplet showing the way that the droplet spreads around the area of the facing gaps. In Figure 4a we also include as an inset the averaged infiltration area which was assumed in the numerical simulations. We note again here that the area responsible for the resonance tuning is the area between the facing gaps, where the high concentration of the electromagnetic fields was found. It is obvious that the presence of the PS shifts the resonance to lower frequencies, $\Delta f_{PS}=\left|f_{PS}-f_{air}\right|$, which is due to the permittivity difference $\Delta \varepsilon_{PS}=\left|\varepsilon_{PS}-\varepsilon_{air}\right|$. The corresponding relative tunability was determined by the relation $RT=\Delta f_{PS}/\Delta \varepsilon_{PS}$. We present the study of the sample with unit cell with size L=5,7,10 mm. In the case of *L* = 10 mm the experimental shift of the resonance was equal to 245 MHz and the corresponding relative tunability was equal to $RT_{L=10\;\mathrm{mm}}=153$ MHz/PU (permittivity unit). For the other cases the relative tunability was $RT_{L=7\;\mathrm{mm}}=400$ MHz/PU or $RT_{L=5\;\mathrm{mm}}=838$ MHz/PU. Although the presented components were not optimized for maximum tunability, it is deducted that the acquired technology may provide a platform for in-house, printed on demand and on-the-fly devices spanning over a large range of frequencies in the microwave regime. Everyday applications involve tunable absorbers, filters, de-multplexers, wavefront controllers, shieldings and many more.

## 4. Summary and Conclusions

We have fabricated metasurface units of conductive SRR configurations using the FDM 3D printing method. The method is low cost, quick and user- and eco-friendly. As spool materials, two different thermoplastic filaments have been used for printing, PLA and Electrifi. The former is a well–known dielectric polymer and therefore the PLA printed SRRs were coated with conductive silver epoxy to obtain high electrical conductivity. The latter is an electrically conductive, polymer-based nanocomposite. SRR structures with varying dimensions and infiltrating material were fabricated and characterized in the microwave regime using standard rectangular waveguides. All fabricated SRRs exhibited well defined electromagnetic responses, which are tunable, with respect to their dimensions and infiltrating materials. Results concretely show that the 3D printed SRR structures exhibit electromagnetic resonance with well defined characteristics. Results also show that the 3D printed SRRs exhibit a sizable resonance shift, with respect to moderate dielectric constant changes. The Fused Deposition Modeling, 3D printing fabrication of SRR-based configurations appears to be an efficient route in the direction of the massive production of high quality, flexible components dedicated for microwave applications.

## Figures and Tables

**Table 1:** Printing conditions for PLA and Electricfy filaments.

Printing Conditions	PLA	Electrifi
Filament Diameter	1.75 mm	1.75 mm
Filament Conductivity	0	$10^{4}$ S/m
Nozzle Diameter	0.4 mm	0.4 mm
Nozzle Temperature	230 °C	140 °C
Printing bed temperature	110 °C	Room temperature
Printing speed	25 mm/s	15 mm/s

**Figure A2:**
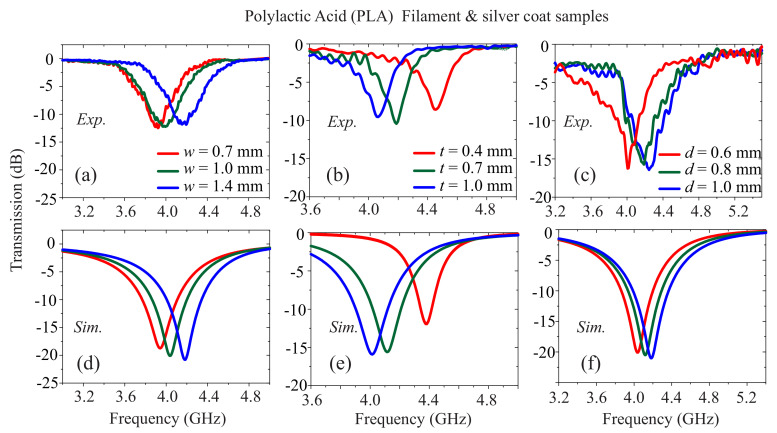
(**a**,**d**) Transmission spectra for PLA/Ag epoxy coated SRRs with various widths. Experimental data (**a**) in comparison with numerical simulations (**d**). (**b**,**e**) Corresponding transmission spectra for SRRs with different wire thickness. Experimental data (**b**) in comparison with numerical simulations (**e**). (**c**,**f**) Transmission spectra for SRRs with various gaps. Experimental data (**c**) in comparison with numerical simulations (**f**). The side length is equal to *L* = 10 mm.

## Appendix A. Design and Characterization Details

Initially we studied the responses of millimeter square, opposing gaps SRRs’ infinite metasurfaces, as shown in the inset Figure A1a. When an electromagnetic wave of suitable frequency and polarization interacts with the SRR, alternating currents circulating in the wires are excited which gives rise to an effective inductive response. On the other hand, the gap and the neighboring metallic elements allow the accumulation of opposite changes which forces an effective capacitance. In all, the SRR behaves as an inductor-capacitor (LC) circuit with the resonance being a function of the shape of the ring, the size and position of the gaps and the details of the impinging wave. At its resonance, the SRR confines the impinging electromagnetic energy at the volume of the gap or between the adjacent meta-atoms. The structure is designed for operation in the low gigahertz range, where we performed the experimental characterizations. For the preliminary numerical design the ring was considered to be made of a high conductivity material ($\sigma =10^{7}$ S/m), whereas no substrate was assumed; that is, the metasurface was free-standing. The overall size of the unit cell, that is, the periodicity of the metasurface, was equal to $a_{y}=2\left(L+w\right)$ and $a_{z}=\left(L+w\right)$ along the *z* and the *y* axis. In the frequency range under consideration E${}_{z}$ polarization obtained a fundamental resonance at 5.2 GHz. In Figure A1a we also present the dependence of the electromagnetic response in variable wire conductivity values in the range $\sigma =10^{3}–10^{7}$ S/m. As observed in Figure A1a (red curves), in the ideal case of $\sigma =10^{7}$ S/m, which is the case of the metals like copper at the microwave regime (fabricated in PCB boards), the response was sharp and the transmission dip was as low as −45 dB. As the conductivity obtained lower values, the response degraded, that is, it became less sharp, the quality factor became smaller, the minimum of the transmission peak was suppressed and the resonance experienced a shift towards smaller frequencies. However, in the case of really substandard conductivity such as $\sigma =10^{3}$ S/m, the resonance was still well defined and quite low (−12 dB) in the free-space infinite metasurface.

We also characterized numerically the electromagnetic properties of the metasurface by placing a single unit cell in the center of a closed rectangular waveguide, and measuring the scattering parameters as shown in Figure A1b. The rectangular waveguide allowed us to trail the resonance of the metasurface in a simple and accessible manner, using a closed system. The approach is valid since the rectangular waveguide mimics the $E_{z}$ polarized TEM mode of the free space planewave which impinges normally the metasurface, and at the same time, it artificially imposes a periodicity which corresponds to a metasurface. In particular, the waveguide supports a single propagation mode in a frequency regime which depends on its dimension, a×b. In the single mode operation the waveguide supports the propagation of only the fundamental TE${}_{10}$ mode (higher order modes decay fast). The single mode frequency range is delimited between the lowest TE${}_{10}$ mode cutoff frequency and the upper TE${}_{20}$ mode cutoff frequency. The spatial profile of the TE${}_{10}$ mode is characterized by no zero crossings; it is polarized in the *z* axis, $E_{z}=\sin\pi /a$, and therefore is as similar as possible to the TEM wave. Additionally, the conditions at the metallic faces of the waveguide are: $E_{z}\left(y=0,z\right)=E_{z}\left(y=a,z\right)$ and $E_{z}\left(y,z=0\right)=E_{z}\left(y,z=a\right)$, and mimic the periodic conditions of the free space metasurface with the restriction of the constant waveguide size with forces an effective periodic unit cell.

Comparing the infinite, free-space metasurface, shown in Figure A1a, and the waveguide simulations shown in Figure A1b, we observe an overall shift of the resonance towards larger frequencies. This is a consequence of the waveguide metallic walls that impose periodic conditions which effectively corresponds to a more sparse metasurface, that is, with larger periodicity. In particular, the periodicity of the infinite, free-space metasurface is equal to $a_{y}\times a_{z}=12.8\times 6.4$ mm and the dimensions of the waveguide are a×b = 35 mm × 15 mm. This practically means that the effective intercoupling between the unit cells is weaker in the waveguide case (and stronger in the free space metasurface where the unit cells are close to each other). Apart from the resonant frequency, this has an impact also on the depth of the transmission peak, which is weaker in the rectangular waveguide. We also presented the conductivity dependent simulation results for the rectangular waveguide study. The effect is the same as in the infinite case. For the high conducting case the transmission dip was −30 dB, and in the case of the low conductivity was as low as −2 dB. Additionally, a small resonance shift with varying conductivity was observed.

## Appendix B. Meta-Atom Response with Variable Geometry

In Figure A2 we study the effect of the geometrical SRR parameters other than the most determining parameter, the side length *L* (presented in the manuscript and Figure 3), on the resonant response of the SRR. The geometrical parameters are found in Figure 2 of the manuscript. Figure A2a,d presents the effect of the SRR width on the transmission resonance (experimental data and numerical data). In particular, we measured the transmission for unit cells with widths w=0.7 mm (red line), 1 mm (dark green line) and 1.4 mm (blue line), respectively (L=10, t=0.4, d=0.6 mm). For the Electrifi conductivity we assumed $\sigma =1.6\times 10^{4}$ S/m, allowing a deviation of 0.5% about the nominal value. The response exhibits only a small dependence on the wire width as the resonance moves towards larger frequencies with increasing *w*. Figure A2b,e presents the effect of the wire thickness in the transmission resonance. In particular, we recorded the transmission for unit cells with variable thickness, t=0.4 mm (red line), 0.7 mm (dark green) and 1 mm (blue line), respectively (L=10, w=2, d=0.6 mm). The response shows a slight dependence on the wire thickness, as the resonance moves towards smaller frequencies with increasing *t*. Finally, in Figure A2c,f we present the effect of the size of the gap. In particular, we measured the transmission for unit cells with d=0.6 mm (red line), 0.8 mm (dark green line) and 1 mm (blue line) respectively (L=10, w=1, t=0.6 mm). The wriggles in the transmission spectra are associated with measurement noise. A slight dependence of the resonance frequency on the gap size was observed, as the resonance curve was shifted to higher frequencies, with increasing gap *d*. This is a consequence of the effective decrease of the meta-atom capacitance which leads to a resonance increase. Once again, we observed an overall experimental resonance with shallower values than the corresponding numerical ones. It becomes evident that the variation of the length *L* of the basic SRR component leads to substantial shift of resonance minima, while all the other SRR parameters (width, thickness, gap size) weakly affect the resonance frequency. Thus, by adjusting the dimensions, especially the length of the SRR, we can regulate the operation frequency of the structure.
